# Factors to improve quality for older patients in the emergency department: a qualitative study of patient trajectory

**DOI:** 10.1186/s12913-021-06960-w

**Published:** 2021-09-14

**Authors:** Isabelle De Brauwer, Pascale Cornette, William D’Hoore, Vincent Lorant, Franck Verschuren, Frédéric Thys, Isabelle Aujoulat

**Affiliations:** 1 Institute of Health and Society (IRSS), Université catholique de Louvain, Brussels, Belgium; 2 Institute of Experimental and Clinical Research (IREC), Université catholique de Louvain, Brussels, Belgium

**Keywords:** Emergency department, Elderly, Process of care, Qualitative observations

## Abstract

**Background:**

Managing older people in the emergency department remains a challenge. We aimed to identify the factors influencing the care quality of older patients in the emergency department, to fine-tune future interventions for older people, considering the naturalistic context of the ED.

**Methods:**

This is a qualitative study of some 450 h of observations performed in three emergency departments selected for their diverse contexts. We performed seventy observations of older patient trajectories admitted to the emergency department. Themes were extracted from the material using an inductive reasoning approach, to highlight factors positively or negatively influencing management of patient’s trajectories, in particular those presenting with typically geriatric syndromes.

**Results:**

Four themes were developed: no geriatric flow routine; risk of discontinuity of care; unmet basic needs and patient-centered care; complex older patients are unwelcome in EDs.

**Conclusions:**

The overall process of care was based on an organ- and flow-centered paradigm, which ignored older people’s specific needs and exposed them to discontinuity of care. Their basic needs were neglected and, when their management slowed the emergency department flow, older people were perceived as unwelcome. Findings of our study can inform the development of interventions about the influence of context and organizational factors.

## Introduction

### Background

Older people (OP) account for 12–24% of emergency department (ED) admissions [1–4]. They have higher ED utilization rates, but this seems appropriate as, inter alia, they consult for more serious conditions than the younger counterpart, and there are barriers to care for those conditions in primary care [1–5]. They are at greater risk of complications after ED visits, e.g. ED readmission, hospitalization, functional decline, institutionalization, and death [1–4]. Moreover, long and complicated histories, the frequent presence of multiple medical co-morbidities, polymedication, and atypical presentations of illnesses (e.g. falls, cognitive disorders) require more time and resources during their ED consultations [1–4].

### Importance

Management of OP in EDs is, thus, a qualitative and quantitative challenge, which means that emergency professionals have to deal with an increasing number of OP, more often suffering from multiples chronic pathologies and atypical presentations of acute diseases [5, 6]. Interventions based on comprehensive geriatric assessment – a multidimensional patient-centered diagnostic process – adapted to the ED context have shown promising results [7–10], but are few [11] and do not seem to be widely implemented. Moreover, they mainly aim to improve care coordination for OP discharged to primary care and do not take into account other dimensions of care quality in EDs [5, 6, 12–14]. Moreover, there are few observational studies of OP care in EDs. To design effective interventions for them, we need a comprehensive exploration of the factors driving care quality for OP in EDs.

### Goals of this investigation

Our study aims firstly to describe procedures for managing older patients in EDs, especially those presenting with typically geriatric issues; secondly, to explore factors that positively or negatively influence care quality in the naturalistic context of the ED.

## Methods

### Study design, settings, and sample

We used a qualitative design suitable to the study of processes and the exploration of mechanisms affecting care quality^16^. The investigation included observations of OP trajectories, in order to describe and analyze the specific process of care for OP in EDs.

The study was conducted in three Belgian sites selected for their diverse contexts. Two were in teaching hospitals in an urban area. The third was in a medium-sized hospital in a region peripheral to this previous urban situation. Each had a geriatric department, making collaboration theoretically possible. We included 3 sites to enrich the observations and not for comparison purposes.

### Data collection

The field researcher (IDB), a geriatrician, conducted observations for 1 month per site on weekdays in the daytime, starting with the first morning shift. Observations focused on management of OP admitted to EDs, involving ED staff, patients, and relatives. They mainly began at the front desk and ended at patient discharge from EDs. The investigator then followed the next older patients admitted at the front desk. When several arrived simultaneously, the researcher chose those with a main complaint not yet observed or a typically geriatric feature.

Field notes were taken, including details of trajectory, geographical details, and care process, as well as personal notes. Ad hoc field interviews supplemented the observation data [15].

### Analysis

Field notes were fully transcribed and analyzed by the researcher who did the observations. She regularly met a senior qualitative researcher (IA) and a geriatric medicine expert (PC) for critical review of the analysis; data were pseudonymized to these experts. Results were then discussed with a panel of experts in emergency care, qualitative analysis, and public health medicine.

All notes were repeatedly read carefully to ensure appropriate content and general understanding. Analysis was then performed in two phases, combining framework and inductive coding. The first phase aimed to identify elderly patients with typical geriatric problems, while the second phase aimed to explore inductively the factors that influence the quality of care for these identified patients.

We based our analysis on Donabedian’s conceptual framework for evaluation of care quality, with a main focus on organizational (as part of structure) and care process, including interaction within and between practitioners and patients [16]. Analysis was based on the observer’s paradigm, a medical intern in geriatrics. Comprehensive Geriatric Assessment (CGA) was thus used as a practical framework for guiding the first phase of analysis. This first phase aimed to identify patients with typically geriatric presentations, i.e. falls, delirium, and functional decline – who are among those who could benefit from CGA in EDs. Indeed, case-finding is the first step of efficient geriatric interventions in emergency department [8]. Analysis of the transcripts involved the systematic search of data related to the broad principles for managing such concerns. Relevant field notes were labelled and classified.

Falls, delirium, and functional decline were analyzed more deeply, as they are frequent reasons for ED admission, are often atypical symptoms of a hidden acute disease, and are likely to predict adverse outcomes in OP [1–4, 17, 18]. Falls included those declared as main complaints and those associated with major somatic complaints in the patient’s history. In addition to age (≥75), risk factors for delirium were reviewed: these included cognitive impairment, assessed through observation of the care process, e.g. interaction between ED caregivers, patients, and relatives, or during consultation of patient files by ED caregivers. Apart from falls and cognitive impairment, other risk factors for functional decline were also noted, including pre-existing functional limitations, advanced age (≥85), being institutionalized, and living alone, especially for people discharged to their own homes.

The second phase of analysis was carried out on the material collected from the patient trajectories identified in the first phase. To develop themes relating to factors that positively or negatively influence the management of these geriatric presentations, we used an inductive reasoning approach [19–21].

At the beginning of the process, some particularly challenging trajectories were coded independently by IDB and IA who met several times to discuss the issues until consensus was reached. The four analytical themes developed were: no geriatric flow routine; risk of discontinuity of care; unmet basic needs and patient-centered care; complex older patients are unwelcome in EDs.

## Ethics

All methods were performed in accordance with the relevant guidelines and regulations. The study, its qualitative method and the written informed consent process with the emergency department Head was approved by the IRB Biomedical Ethics Commission Hospital-Faculty of UCLouvain (2010/25jui/81). The ethical officials of the three anonymized hospitals in which the observations were conducted gave their written approval to the study.

The study used naturalistic observations conducted in collectivities, i.e. emergency departments. This implies adaptation of ethical consent [22–24].

First, an informed consent form has been submitted to the relevant institutional officials following recommendations for naturalistic observation of a process. Written informed consent was obtained from the ED head, as representative of the emergency department staff, after a presentation of the study to the staff of the departments, as a group.

Second, the researcher (IDB), a geriatrician, was an overt non-participant. Professionals, patients, and/or relatives were informed about the researcher’s role; a written research summary, including contact information, was available on request. Verbal consent was systematically and directly requested by IDB from all those present during the study, including ED staff, patients, and their family/carers, prior to observation. Contact information and prolonged immersion in the wards allowed to create the conditions to exercise free informed consent at any time. This respects the key principle of human autonomy. Moreover, potential harms arising from participation in the research were considered as no greater than those arising under usual practice.

Specifically, and although they were not direct subjects of the research, the patients were an important concern throughout the observation process. We supported a not strictly normative ethical attitude, which sought to preserve the principle of autonomy and non-maleficence towards patients. IDB therefore systematically informed patients about her presence, the aim and modalities of the study, answered any questions, asked for verbal consent, and made her contact details available. When patients suffered from overt cognitive impairment, this process was performed with a relative, present on site. If this could not be achieved, the observations were directed towards the professionals rather than the patients themselves, aiming to respect their intimacy, while not soliciting them for the research (*primum non-nocere*).

Finally, all data were pseudonymized and remained confidential.

## Results

### Characteristics of study subjects

We observed 70 cases over 450 h. For patient characteristics, see Table 1. Some observation details are summarized in Table 2. Lengths of stay ranged from 0.75 to over 32 h and involved 2 to 12 ED caregivers and/or consultants. Forty-eight patients (69%) were hospitalized.

**Table 1:** Patients’ characteristics (*n* = 70), mean (range) or number (%)

Age, years	82 (72–96)
75 and older	68 (97)
Sex (female)	47 (67)
Residence	
Community-dwelling	49 (70)
Nursing home	18 (26)
Rehabilitation center	1 (1)
Unknown	2 (3)
Presence of a relative	40 (57)
Community-dwelling	31
Nursing home	7
Unknown	2
Referred by a physician	37 (53)
Own general practitioner	24
Another general practitioner	5
Specialist in the institution	6
Outside specialist	2, including 1 from rehabilitation center
Referred by a caregiver, with a written note	32 (46)
Own general practitioner	17
Another general practitioner	5
Outside specialist	1, from rehabilitation center
Nursing home	9, including 7 not referred by a physician
Falls, recent history	24 (34)
Main reason for admission	17
Accompanied somatic chief complaint	7
Delirium, > = 2 risk factors	61 (87)
Pre-existing cognitive disorders	18
Neurological main complaints	9
Deterioration of general status	12 (17)

**Table 2:** Observations: details

Hospital 1	Hospital 2	Hospital 3
34 observations	16 observations	20 observations
Approximate mean length of stay: 4 h	Approximate mean length of stay: 7 h	Approximate mean length of stay: 6h30m
Approximate mean number of caregivers that intervened in the process of care: 4 • ED, MD trainee: 0 • ED, MD assistant: 1/3 • ED, MD: 1 • ED, GP: 2/5 • in-hospital, MD: 1 • ED, Nurses: 2	Approximate mean number of caregivers that intervened in the process of care: 7 • ED, MD trainee: 1 • ED, MD assistant: 2 • ED, MD: 1 • ED, GP: 0 • in-hospital, MD: 2 • ED, Nurses: 2	Approximate mean number of caregivers that intervened in the process of care: 5 • ED, MD trainee: 2/5 • ED, MD assistant: 1 • ED, MD: 0 • ED, GP: 0 • in-hospital, MD: 1 • ED, Nurses: 2

*ED* emergency department, *MD* medical doctor, *GP* general practitioner.

### Main results

Almost all the patients would have benefited from screening for cognitive status, functional performance, and/or psychosocial support to highlight hidden, typically geriatric problems. Indeed, two or more risk factors for functional decline were noted in 58 of the 70 (83%) patients, while 63 (90%) presented with at least one of the three typical geriatric presentations, i.e. falls (*n* = 24, 34%), high risk of delirium (*n* = 61, 87%), and functional decline (*n* = 12, 17%) (Table 1). The trajectories of these 63 patients were taken into account in the second stage of the analysis.

### No geriatric flow routine

Overall, the patients received the same care process. Their management was mainly based on a systematic biomedical approach. For example, when Dr. B had an elderly patient with deterioration in general status [similar to functional decline, Ed.], she always requested a radiographic examination of the thorax and laboratory tests (Observation notes H1–2).

ED caregivers generally had no triage routine to screen for a geriatric profile, including functional status, and identify older patients at risk of complications. One hospital had incorporated such a process, using a screening tool, into the nurse’s triage but implementation was almost never done: *“I always skip it [screening]; it is in the wrong place and should be part of triage, and I forget it. Whereas charging, we’ve been drilled, we don’t forget it.”* (H3–48, nurse’s interview notes); *“there are more urgent things; I forget items; it’s not difficult, but going through it every time, ugh!”* (H3–51, nurse’s interview notes). This highlights the low priority given to geriatric “triage” compared to other, purely technical and financial tasks. In particular, systematic screening for delirium and subsequent prevention measures were not part of standard procedures. However, repeated screening of elderly people for delirium, starting at triage, is of paramount importance, as it is a frequent and serious geriatric syndrome: precipitating factors must be rapidly assessed, and specific care and monitoring put in place to limit the consequences. Patients with a geriatric profile require a specific management, because of its inherent risk of adverse outcome following ED consultation and acute admission.

Management followed two guiding principles, prioritization and categorization, to label the problem presented. Prioritization assigned an acuteness level; categorization specified the nature of the priority. This was to exclude organic health conditions requiring rapid treatment, e.g. hip fractures, and to maintain patient flow. In some cases, procedures requiring a prescription were initiated by a nurse, *“to save time because we know what the doctors want”* (H3–52, nurse’s interview notes). Prioritization and categorization at the triage stage were of paramount importance for subsequent management. For example, a patient (H1–10) admitted by ambulance was not triaged by an ED nurse but sent directly to trauma for a fall, delaying treatment of an underlying heart problem. Moreover, when falls were the main reason for admission (*n* = 17), they were systematically labelled as traumatic issues. Preoperative assessment in 6 of these cases ruled out any urgent underlying somatic cause. However, minor trauma, particularly uncomplicated wounds, did not automatically lead to such assessments: management focused on the primary motivating category, e.g. “wound requiring suturing”. However, falls are also an important geriatric concern, as they are often an atypical presentation of an underlying medical problem to exclude. Being triaged and labelled as a “simple wound to repair” expose patients to incomplete evaluation and possible inadequate primary care and/or geriatric follow-up.

Categorization also allowed implementation of a treatment plan, in particular to facilitate “negotiation for a bed” with a consultant, where necessary. A bed then became the priority. One of the hospitals allocated one nurse to this. Consultants contacted during the care pathway were mainly organ specialists, corresponding to the categorization. However, categorization in a specific specialty was often a challenge for multimorbid patients. Additional technical examinations were regularly used to facilitate negotiation for a bed. Consultants often set “technical” conditions for admission; this sometimes prolonged ED consultations, slowing down the flow and efficiency, with little added value for patients as explained by Dr. J.: *“So Dr J. phoned the lung specialist. The doctor she spoke to wanted an echocardiogram. I was surprised and asked Dr J why. She said she didn’t know why but she was doing it because she might lose out on a bed. She dressed the patient’s story up for the ultrasonographer, who agreed to conduct the examination.”* (H1–8 observation and interview notes).

A geriatrician was called in for 12 of the 63 patients presenting with one of the three geriatric issues (19%), mainly to obtain admission to the geriatric ward, not for a specific advice for the management of these issues within the ED. Four of these patients already had a link with that department; six others had psychosocial issues and presented with deterioration in general status. The lack of beds in geriatrics was often mentioned, which could be one reason why a geriatrician’s advice was not sought: *“There is definitely no bed in the geriatric department. By the way, there’s never any bed in the geriatric department! Many people are not treated in the right department.”* (H3–47 nurse’s interview).

### Risk of discontinuity of care

An average of five caregivers intervened per patient, including ED team members and others from other specialties. ED caregivers, moreover, changed during shift handovers and each was often responsible for several patients with different problems, priorities, and, possibly, very different timeliness. A high number of caregivers and care recipients could expose to discontinuity of care and loss of information. The case of Mr. F., aged 81, admitted from a rehabilitation center for a fall with a complicated wound (H3–55), illustrated the risks of this division of work for both care quality and ED flow. A 27-h stay, intervention by at least 12 professionals, and unclear leadership illustrated the difficulties of passing on the information needed to bring the pathway to an end. In some cases, the observer was approached by ED caregivers, mainly with regard to continuity of information. Observation of a self-appointed case-manager illustrated the importance of coordination of the care process in EDs: a woman aged 81 (H1–12) arrived with a cardiac rhythm problem at a busy time. Her grandson, an emergency nurse assigned to triage, acted as case-manager until she returned home, while complying with the established prioritization rules. He ensured that information was passed on within the team and to the patient, identified the additional examinations needed, and the responsible physician. Eight professionals were involved in this pathway, even though she only stayed for about 3.5 h.

### Unmet basic needs and patient-centered care needs

Functional status rarely figured in the medical history or ED assessment. It was addressed in 9 of the 63 cases by an ED caregiver, including one social worker and one ambulance doctor, and spontaneously declared by the patient or a relative in two. Medico-technical care was the priority, often at the expense of traditional bedside and patient-centered care. Some ED caregivers managed to reconcile technical care with a more global approach. Nevertheless, these kinds of care were in competition, given the irregular flow of patients, which hindered efforts to meet basic needs such as comfort, pain relief, food, or hydration: *“I would like to put her in a bed because she’s in such pain ( …*), *but I still have two infusions to set up.”* (nurses’ observation notes H1–6).

Two ED caregivers were particularly attuned to these basic needs. They had experience with a different care paradigm. A social worker provided in-home coordination that took account of the patient’s functional status and preferences (H1–3, observations). One nurse – who had worked on a pediatric ward – was particularly attuned to patients’ basic needs (H1–6): she was quite respectful and took the time to explain the steps. She put a cover over her, ensured she was comfortable, and tried to reassure her. She talked about her previous experience, which wasn’t typical for an emergency nurse … *“I like the [observation unit] and reception. I pamper my patients. Most here have flashing lights on their heads!”* [They prefer emergency ambulance callouts and management of critically ill and polytrauma patients, etc.] (H1–6 observation notes and spontaneous nurse’s declaration).

Furthermore, management was carried out without consulting older people and considering their priorities, which led to unexpressed and therefore missed needs. They were deemed to have agreed to the process, without consulting them for approval about management. Although rarely involved in decisions, they seldom complained about this. But they sometimes addressed information requests and basic care concerns to the observer. A relative can be a precious resource, mainly as companionship for the patient, for communicating basic needs and transmitting information. For patients with cognitive disorders, conclusions and treatment plans were entrusted to a relative. In one case, daughters adopted a more proactive attitude, disagreeing with the doctor’s conclusion that “the assessment had turned up nothing unusual” and with “the decision to send the patient back [to the nursing home] without any explanation [of the symptoms] …. They requested a second opinion …! Dr. Y … contacted the geriatric department. He was not convinced that hospitalization was justified, even if he sympathized with the daughters. (H2–36, observation notes), highlighting an emotional dimension of the evaluation.

### Complex older patients are unwelcome in EDs

During certain observations, the issue of caring for elderly patients was raised with ED professionals.

Its legitimacy was almost never questioned at the front desk or at the triage step, or at least the elderly patient was not blamed. They were often legitimized by their general practitioner’s referrals and stereotyped in such a way that if they present at ED, it is because they could not do otherwise, that they do not abuse the system: *“They have often called the family doctor [unlike younger patients]. If they come to the hospital to be admitted [to a hospital care unit], it is often at the request of the family, who want to get rid of the parents.”* (H1–11, interview notes) *“Some people call an ambulance to jump the queue – young people, not older people. Older people seem ‘stronger’.”* (H2–22, summary notes, nurse’s interview) Moreover, prioritization seemed to be easier for older people *“because either they [older people] arrive by ambulance and are by definition priority or they are accompanied.”* (H1, triage secretary interview).

However, during subsequent stages, old age was often associated with complexity of care – one caregiver even referred to troublesome cases (H3–55 observation notes) – and/or slowness, things that do not fit the current emergency department care model (H2–43 interview Dr. M). *“We don’t have much time, but they take up a lot of time even though there are so many people [in the waiting room]”* (H2–43, nurse’s interview). *“They block up the emergency department”* (H3–47, nurse’s interview). *“I don’t like geriatrics … [in the ED] you have to work quickly and nothing is simple with elderly patients.”* (H1–7, interview Dr. G).

The feeling that older patients are a burden was indirectly illustrated by overestimations of their proportion in EDs (H2–42, caregiver’s interview; H3–43, caregiver’s interview). One caregiver described his/her distress when admitting older patients in the ED observation unit, saying [he/she] was incapable of taking care of them...and needed help *“if one weeps and cannot cope”* (H2–42, Dr. AI).

Although the legitimacy of presenting at the department was rarely questioned directly, two doctors criticized the failure of care in nursing homes. One described a case as “prophylactic” and could not *“understand why this patient has been sent in [before the weekend], especially since she has come from a nursing home”* (H3–50, observation notes Dr. AC); the second observed: *“when elderly people come in at night, it really is serious … or it’s a nursing home that sends them in because they can no longer cope.”* (H2–24 interview notes, night-shift doctor).

These attitudes may be associated with stereotypes about elderly patients, which may explain, e.g., a caregiver mainly addressing a daughter before realizing that the patient was cognitively sound (observation notes H2–45). One professional ignored the patient and seemed uninterested in treating the elderly (observation notes H2–40). This recalls ED caregivers’ problems with consultants reluctant to admit elderly patients, as they want patients to fit their specialty’s parameters (general observation notes H1 and observations H1–2, H2–22, H2–38, H3–65).

## Discussion

For many years now, several projects have sought to meet the challenge of treating the elderly in emergency wards. The findings of this empirical study describe processes that may put OP at risk and factors that influence care quality in EDs. This knowledge is important to fine-tuning ED procedures for OP, considering the naturalistic context of the ED.

Our results raise important concerns: while nine out of ten patients had at least one typical geriatric issue, the technical and highly specialized management process in the emergency department did not meet their specific needs, even the most basic ones, highlighting some thorny issues, particularly that of neglect.

The four themes discerned in their management were similar in the three hospitals: no geriatric flow routine, risk of discontinuity of care, unmet basic needs patient care needs and, finally, complex older patients are unwelcome in EDs.

First, although almost all patients would have benefited from screening for psycho-cognitive and functional status, procedures were not adapted to their assessment. Emergency caregivers adopted a sort of batch processing, performing multiple tasks for which they followed set categories and priorities, based on biomedical considerations. They aimed to exclude organic conditions that could be treated quickly and to maintain patient flow, including finding beds for some. As older patients were often hospitalized, the lack of beds was possibly one factor in the limited contact with geriatric departments. Triage was of particular importance, as it determined subsequent management. At the start of triage, the biomedical care process applied was inadequate to ensuring quality of care for older patients, particularly those presenting with geriatric issues: falls were not systematically recognized as possible consequences of underlying medical problems and delirium was not screened. Failure to recognize these syndromes could mean unmet needs and further adverse outcomes, a matter of concern in the literature [1–4]. More generally, screening for frailty – geriatric triage – has been shown to increase the effectiveness of geriatric interventions designed to prevent adverse outcomes after ED admission [8, 10]. The issue of reconciling care quality and organizational efficiency, especially patient flow, has been discussed by previous authors [25–28], who showed how doctors in emergency departments have to manage apparently contradictory concepts: clinical, organizational, and social aspects and policy directives. These tensions are particularly important in emergency departments. EDs are available 24/7 and admit a potentially unlimited number of patients, with unpredictable health problems of all kinds; they are also a hub for access to the health care system, particularly as a front door for admission to acute hospitals with limited capacity. Although “flow culture” is of paramount importance for efficiency, it can lead to reduced adherence to guidelines and screening routines [29].

Second, a great many caregivers intervened during older patients’ care pathways; specialized skills and expertise imply a division of labor. Our results showed that lack of coordination exposed OP to care fragmentation and information loss. As care fragmentation is a well-known source of medical errors and negative experiences of emergency department care [30, 31], our findings raise concerns. Conversely, teamwork, including coordination and communication, helps to improve patient safety and patient and staff satisfaction and reduce medical errors. A Canadian coordinated nurse-led care project has been described, but not yet studied in terms of patient and health system outcomes [32].

Third, the priority given to medico-technical care was often at the expense of patient-centered and bedside care, a conflict already described by some authors [14, 33]. Failure to meet these needs is definition of neglect [34]. Those few caregivers who were aware of the issues in caring for older patients had experience of a more global care paradigm. As populations age, this raises the question of whether ED organization is still adequate. Innovative solutions have been suggested with a view to fine-tuning the current care model [5]. These involve profound changes in the current health care system paradigm, in both hospitals and primary care. Another issue is the opportunities offered patients to participate in their treatment. These are almost nonexistent, but older patients generally did not complain about this. This is worrying, as participation has been shown to improve both care quality and patient satisfaction [13, 30, 35–38]. In our study, relatives sometimes played an advocacy or even surrogacy role by communicating patient needs; they were also entrusted with the conclusions and treatment plan when patients with cognitive disorders were discharged. The role of family caregivers for older relatives has been recognized internationally, in relation to coordination tasks, quality-of-life issues, and economic concerns [39, 40].

Fourth, the burden of treating older patients who don’t fit the usual ED care process was often raised; they were described as bed-blockers and unwelcome patients by some caregivers. OP legitimacy is the subject of previous studies, in light of the tension between flow management and clinical, organizational, and social categorization [25, 26, 28]. Legitimacy was rarely questioned at the triage stage, as they are deemed to present acceptable medical and moral justifications for their ED attendance. Once their management blocked the flow, however, older people often became undesirable [25, 26, 28]. Geriatric care is complex: it challenges the usual ED care paradigm. As populations age, EDs will face increasing demands for acute decompensation of chronic diseases and health problems requiring urgent intervention. Care issues may lead to stereotyping, with the risk of attitudes developing that may reduce care quality [12, 41–43]. Improving caregivers’ training via hands-on approaches is one way to develop more confidence when dealing with older people, but would probably be insufficient [5, 6, 12, 44, 45]. Other proposed strategies include awareness-raising among caregivers of their susceptibility to stereotypes and inappropriate attitudes [46].

## Limitations

Limitations are discussed following Lincoln and Guba’s criteria [47]. The observations were conducted by a single researcher with a medical background, including work in one of the ED sites during her internship. While this familiarity facilitated collecting and understanding of data, it may have distorted the findings: firstly, she may have been less sensitive to things that would be noticed by non-medical investigators; secondly, she could be constrained by loyalty. The study’s credibility, however, was reinforced by observing diverse cases over a long period with total immersion in care teams. In the same way, this immersion allowed the researcher to be forgotten as such and to become part of the team, limiting the impact of her presence on the usual management of the patients. Even if iterative process between observations and data analysis was not done during the observations, this was compensated for by comparison between trajectories and discussion between researchers in order to increase the credibility of the results: the notes were analyzed by the field researcher with the aid of the second and last-named authors: one expert in geriatrics and gerontology, who did a PhD about ED settings, and one in qualitative methods, with considerable expertise in health care research. The inclusion of an ED professional at this stage would have been an added value. Nevertheless, discussions with all the experts included in the authorship provided a critical appraisal of the observations. In addition, the validity of the study relied on triangulation with interviews and the large number of cases. Moreover, we can argue that descriptive saturation was achieved since the themes developed from the latest data showed no new themes compared to those developed from the first data collected. The issue of transferability should also be mentioned. The study was conducted in three French-speaking Belgian sites, selected for their diversity, with a long period of immersion. The theoretical framework and the description of the general environment and culture may be easily transferred to other EDs and help international readers recognize similarities. The results could also be transferred to populations displaying similar medical and psychosocial issues.

## Conclusions

Our observations provide a unique empirical insight into management of OP in EDs, including the different actors intervening, while bearing in mind the difficulties of the ED environment. Awareness of the specific presentations of OP, early in the triage stage, would make it possible to better categorize the problems in question. Furthermore, it would make it possible to anticipate and set in motion specific “geriatric” processes and procedures to meet both the needs of elderly people and the requirements of patient flow. Reducing inappropriate ED admissions by early identification of frail patients and care planning in view of their needs and preferences could be an option, but would be insufficient and would risk discrimination against OP. The acute care process should be adapted to this population, at greater risk of admission than others. Particular attention should be paid to care for OP, to prevent negative images and inappropriate attitudes in EDs. Acknowledging that ED organization is unsuited to OP needs and the introduction of specialized caregivers may improve the overall process. Such changes go beyond the emergency setting, however, and should extend to the entire care network and policy for OP.

## Data Availability

The datasets generated and analysed during the current study are not publicly available due to confidentiality of participating hospitals (see “Ethics approval and consent to participate”). Data are however available from the authors upon reasonable request and with permission of the participating hospitals.
